# Thermal and Fluid Dynamics Performance of MWCNT-Water Nanofluid Based on Thermophysical Properties: An Experimental and Theoretical Study

**DOI:** 10.1038/s41598-020-62143-3

**Published:** 2020-03-20

**Authors:** Zongjie Lyu, Amin Asadi, Ibrahim M. Alarifi, Vakkar Ali, Loke K. Foong

**Affiliations:** 1 Institute of Research and Development, Duy Tan University, Da Nang, 550000 Vietnam; 2 Faculty of Civil Engineering, Duy Tan University, Da Nang, 550000 Vietnam; 3 Department of Mechanical and Industrial Engineering, College of Engineering, Majmaah University, Al-Majmaah, 11952 Riyadh Saudi Arabia; 4 Engineering and Applied Science Research Center, Majmaah University, Al-Majmaah, 11952 Riyadh Saudi Arabia; 5 Department for Management of Science and Technology Development, Ton Duc Thang University, Ho Chi Minh City, Vietnam; 6 Faculty of Civil Engineering, Ton Duc Thang University, Ho Chi Minh City, Vietnam

**Keywords:** Chemical engineering, Mechanical engineering, Other nanotechnology

## Abstract

There are many debates on the preparation methods and the role of ultrasonication on the stability, thermophysical properties, and heat transfer performance of nanofluids. The present study, which is the continuation of the authors previous study, the effects of ultrasonication on the thermal and fluid dynamic performance of MWCNT-water nanofluid, over a different range of temperatures and solid concentrations, based on the thermophysical properties of the nanofluid, has been investigated. The effects of ultrasonication time on the stability and thermophysical properties of the nanofluid were studied over 30 days of the samples preparation. The thermophysical properties of the nanofluid have been experimentally measured at the optimum ultrasonication time. Using the experimental data, and employing different figures-of-merit, the effects that the addition of MWCNTs had on the heat transfer effectiveness and pumping power have been studied. It was confirmed that the nanofluid is a good heat transfer fluid, with a negligible penalty in pumping power. The thermal and fluid dynamic performance of the nanofluid in a microchannel heat sink has also been studied, by comparing the enhancement ratio of the convective heat transfer coefficient and the increase in pumping power.

## Introduction

It is well known that the process of energy transport has been integrated into different industrial applications. As a result, researchers have focused on developing more efficient and compact heat transfer facilities over the past decade and a half. Thus, various methods have been investigated to achieve this target, such as the extension of heat transfer surfaces (micro and minichannels), modifications of materials, optimization of the effective parameters, and so forth. Moreover, many researchers have aimed to improve the heat transfer performance (HTP) of the conventional working fluids, such as water, ethylene glycol (EG), glycerol, thermal oils, etc. In this regard, Choi and Eastman^1^ introduced the term ‘*nanofluid*’, which is a mixture of nano-sized particles in conventional working fluids. Research has also focused on thermophysical properties^2–4^ and heat transfer of nanofluids^5–11^, and the feasibility of their use in practical applications^11–17^. Morover, many research has been done on the aplications of artificial intelligence for predicting the thermophysical properties of nanofluids^18–22^. Additionally, in recent years, there have been many reviews of the thermophysical properties^23,24^, modeling and simulation methods^25,26^, and HTP of different nanofluids in practical applications^27–31^.

The convective heat transfer coefficient (CHTC) of nanofluids is the most important factor in heat transfer applications. Moreover, the effective thermophysical properties of nanofluids have an effect on the CHTC. Among the thermophysical properties, thermal conductivity is an indicator for the heat transfer effectiveness, dynamic viscosity is directly affects the pressure loss and pumping power (PP), and specific heat indicates the ability of the working fluid to store and move the generated heat away from the hot source. Thus, measuring or predicting the thermophysical properties of nanofluids is the first step towards evaluating their HTP in practical applications^32,33^. However, it has been proven that the classical models are not able to make a good estimation of the thermophysical properties of nanofluids, within the acceptable range of accuracy^34–36^.

The available literature of rheological properties of nanofluids showed that adding nanoparticles to different base fluids increased the dynamic viscosity^37,38^. However, there were some exceptions, which reported that adding extremely low solid concentrations of MWCNT nanoparticles (0.1 wt %) decreased the kinematic viscosity of MWCNT-oil nanofluid^39^. The general reported trend for thermal conductivity in the available literature showed that adding nanoparticles that possess higher thermal conductivity compared to the base fluid increased the thermal conductivity of the base fluids. Moreover, it was reported that an increase in the temperature also increased the thermal conductivity of nanofluids^40–43^. The primary reason for this was because an increase in temperature leads to an increase in Brownian motion, which is the random motion of particles. This was reported as the main responsible mechanism for increasing the thermal conductivity by increasing the temperature^44^, although, other mechanisms exist as well^45,46^.

It is evident that thermal conductivity and viscosity of nanofluids play a pivotal role in the HTP of nanofluids. However, conducting experimental studies are costly and time-consuming. Thus, prior to conducting an experimental study on the HTP of a nanofluid, it is crucial to make sure that the prepared nanofluid would provide advantages as a heat transfer fluid. To do so, there are figures-of-merit (FOM) which can be used to evaluate the capability of the nanofluids as a heat transfer fluid, based on their thermophysical properties. Asadi^47^ proposed a guideline to estimate the HTP of a nanofluid, before conducting heat transfer experiments. It was suggested that after measuring the thermophysical properties of a nanofluid, employing the Prasher *et al*.^48^ and *Mo* number^49^ FOM, the HTP of the nanofluid should be evaluated for both the fully developed internal laminar and turbulent flow regimes. Cabalerio *et al*.^50^ have also studied the HTP of water-EG (90–10%) nanofluid containing graphene nanoplatelets over different ranges of solid concentrations and temperatures. They measured the dynamic viscosity and thermal conductivity of the nanofluid. Using the experimental data, they then evaluated the HTP of the nanofluid. For the fully developed internal laminar flow, they used the Prasher *et al*.^48^ FOM, and the *Mo* number^49^ as a FOM for evaluating the HTP of the nanofluid in the fully developed internal turbulent flow regime. They reported that the studied nanofluid would be advantageous in the fully developed internal laminar flow regime, but not in the fully developed internal turbulent flow regime. They also considered the effects of adding nanomaterials to the base fluid on the PP and pressure loss and reported that some penalties would be experienced by adding the nanoparticles. In another study, Zyla^51^ investigated the HTP of MgO-EG nanofluid at different temperatures and solid concentrations based on Prasher *et al*.^48^ and *Mo* number^49^ FOMs. There are a limited number of studies in the current literature on the HTP of nanofluids, based on different FOMs, before conducting experimental tests on heat transfer. A summary of the published papers has been presented in Table 1.

**Table 1:** A summary of the available literature on the HTP based on different FOMs.

Studied NF	Temperature range	SC	Remarks	Reference
Fe_2_O_3_-water	10–70 °C	5–20 vol. %	The dynamic viscosity and thermal conductivity of the nanofluid were experimentally measured. The HTP was evaluated, based on the *Mo* number^49^. It was reported that using the nanofluid instead of the base fluid was advantageous in laminar flow, but not in the turbulent flow regime.	Colla *et al*.^74^
Al_2_O_3_-MWCNT/oil	25–50 °C	0.125–1.5 vol. %	The HTP of the nanofluid was evaluated, based on the Prasher *et al*.^48^ and *Mo* number^49^ FOMs for the internal laminar and turbulent flow regimes, respectively. This was based on the experimental data of thermal conductivity and dynamic viscosity. It was reported that using the nanofluid was advantageous in internal laminar flow for all the temperatures and solid concentrations, while it was beneficial to use the nanofluid in solid concentrations less than 1 vol.%, in internal turbulent flow regime.	Asadi *et al*.^3^
ZnO-EG/water	283.15–343.15 K	0–5 vol. %	The dynamic viscosity and thermal conductivity were experimentally measured and based on the experimental data; the HTP was evaluated. The *Mo* number^49^ was used as a FOM, to evaluate the HTP before performing experimental tests on heat transfer. It was found that the nanofluid was beneficial in both the internal laminar and turbulent flow regimes.	Cabaleiro^75^
Mg(OH)_2_-MWCNT/oil	25–60 °C	0.25–2 vol. %	Prasher *et al*.^48^ and *Mo* number^49^ FOMs were employed to evaluate the HTP of the nanofluid in the laminar and turbulent flow regimes, respectively.	Asadi *et al*.^33^
GOnPs/EG-water	283.15–343.15 K	0–0.5 vol. %	Considering the experimental data of dynamic viscosity and TC, the HTP in both the internal laminar and turbulent flow regimes was evaluated, using Prasher *et al*.^48^ and *Mo* number^49^, respectively. The PP was also evaluated, based on a FOM.	Cabaleiro *et al*.^50^
ZnO-oil and MgO-oil	15–55 °C	0.125–1.5 vol. %	The HTP for the laminar and turbulent flow regimes were evaluated, considering the experimental data of thermophysical properties.	Asadi and Pourfattah^32^
MgO/EG	298.15 K	0–20 vol. %	The HTP of the MgO-EG nanofluid were evaluated according to the Prasher *et al*.^48^ and *Mo* number^49^ FOMs, using the experimental data of thermal conductivity and dynamic viscosity.	Zyla^51^
MgO-MWCNT/oil	25–50 °C	0.25–2 wt. %	The HTP of the nanofluid in different temperatures and solid concentrations was evaluated for the laminar and turbulent flow regimes, employing the Prasher *et al*.^48^ and *Mo* number^49^ FOMs.	Asadi *et al*.^12^
Al_2_O_3_-water and Al_2_O_3_-polyalphaolefin	298.15 K	1–5 vol. %	The HTP of the nanofluids were estimated using different FOMs under three different constraints: constant Reynold number, flow rate, and PP.	Yu and Liu^76^
MWCNT-ZnO/oil	15–55 °C	0.125–1 vol. %	The HTP was evaluated, based on the rheological and thermophysical properties of the nanofluid for different temperatures and solid concentrations.	Asadi^47^

It is also important to evaluate and compare the thermal and fluid dynamics performance of nanofluids with those of the base fluids. Generally, in experimental investigations on convective HTP of nanofluids, the performance efficiency coefficient (PEC) is defined as the ratio of heat transfer enhancement to pressure loss. A ratio higher than 1 indicates that using the nanofluid is advantageous over the base fluid; the higher the PEC, the better the HTP. However, it is possible to perform such an evaluation before conducting the experimental tests.

From the literature that has been discussed thus far, it can be concluded that the thermophysical properties of nanofluids play a significant role on the HTP and PP in practical applications. Moreover, evaluating the possible advantages and disadvantageous of using a nanofluid instead of the base fluid, before conducting experimental tests, may prevent excessive costs in performing unnecessary experiments. To the best of the authors’ knowledge, a comprehensive investigation has not been done, thus far, on the effects of ultrasonication time on the thermal and fluid dynamic performance of MWCNT-water nanofluid. Thus, in the current study, the effects of adding MWCNT into water, and the ultrasonication time, on the thermal effectiveness and PP over different ranges of temperature and solid concentrations, in fully developed internal laminar and turbulent flow regimes, will be investigated. The thermal and fluid dynamics performance will be evaluated, and the results will be presented and discussed.

## Materials and methods

### Sample preparations and stability analysis

**Figure 1:**
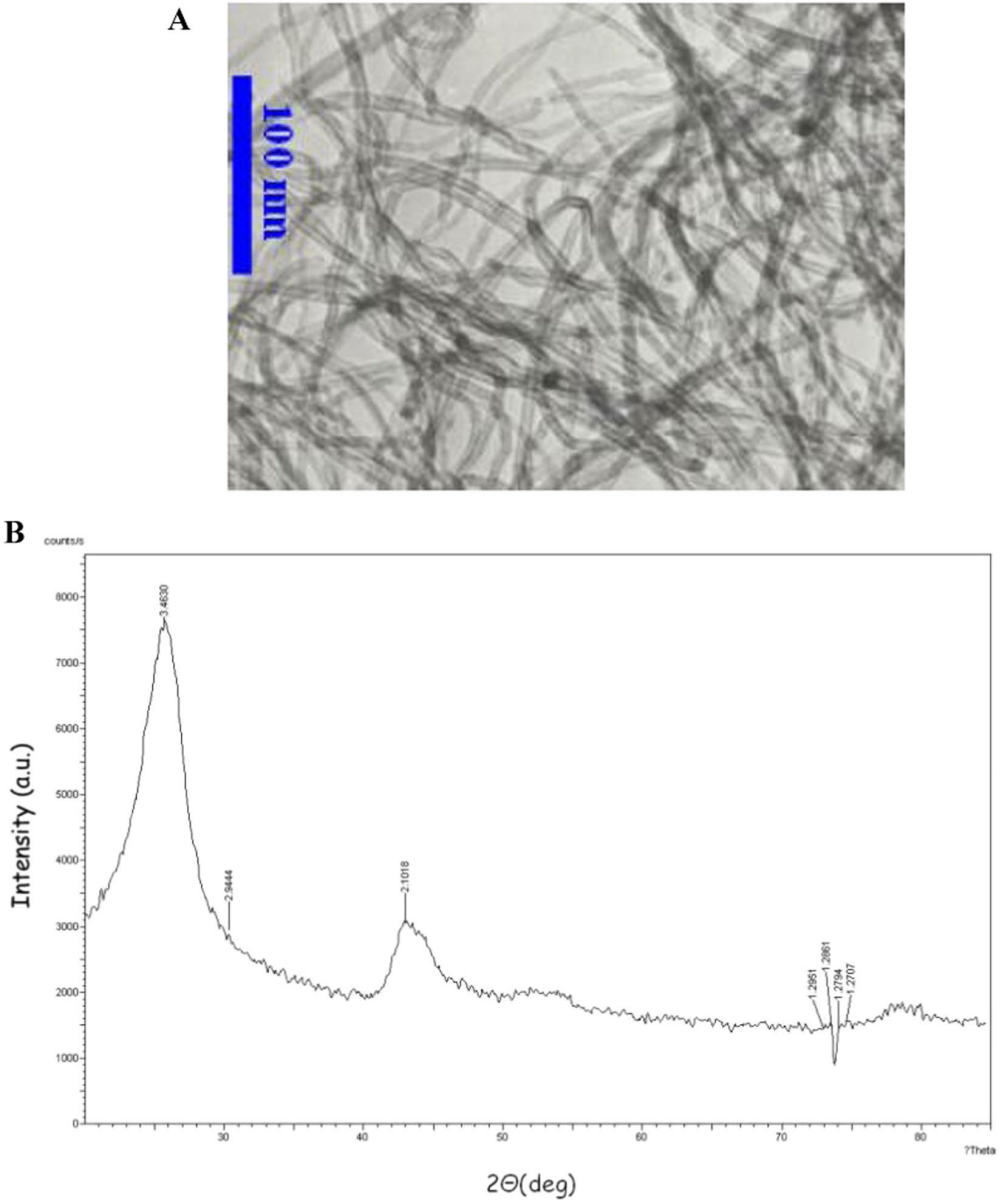
(**A**) TEM image and (**B**) XRD graph of MWCNT nanoparticles^54^. (Reprinted with the permission of Elsevier).

**Figure 2:**
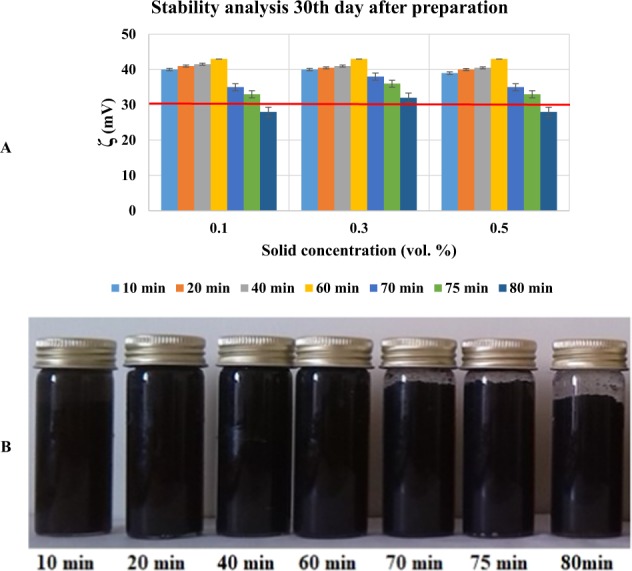
Results of the stability analysis. (**A**) Zeta potential analysis versus solid concentration for different ultrasonication times, and (**B**) visual observation versus ultrasonication time for a solid concentration of 0.5 *vol*. %^54^. (Reprinted with the permission of Elsevier).

In this study, MWCNT nanoparticles (detailed information of the nanoparticle has been presented in Table 2) have been dispersed into water, which was chosen as the working fluid. Figure 1 displays the transmission electron microscopy (TEM) and X-ray powder diffraction (XRD) graphs, which are provided by the manufacturer of the nanoparticles (US Research Nanomaterials, Inc., USA). The samples were prepared utilizing the two-step method, which is a widely used method in the literature^52,53^, in three different solid concentrations: 0.1, 0.3, and 0.5 *vol*. %. Detailed information about the preparation process can be found in ref. ^54^. Different ultrasonication times were applied to disperse the nanoparticles and break down the clusters of nanoparticles into the base fluid. This was executed using a probe-type ultrasonic device (20 kHz, 1200 W) because the probe/horn ultrasonic devices are more effective than water-bath ultrasonic devices^55,56^. The effects of ultrasonication time on the stability of the samples were studied by conducting a visual observation (as a quantitative method) and zeta potential (ζ) analysis (as a qualitative method) over 30 days, after the preparation of the samples. Other literature indicated that suspension with a ζ higher than +30 *mV* and −30 *mV* provides good stability for a long period^57,58^. The results showed that 60 min ultrasonication is the optimum time, for which the samples are the most stable, even after 30 days of preparation. Prolonging the ultrasonication resulted in the deterioration of the sample quality. Figure 2 shows the result of zeta potential and visual observation after 30 days of preparation.

**Table 2:** Detailed information of MWCNT nanoparticles^54^. (Reprinted with the permission of Elsevier).

Outside diameter	<7 nm
Inside diameter	2–5 nm
Length	10–30 um
SSA	>500 m^2^/g
Electrical conductivity	>100 s/cm
True density	2.1 g/cm^3^
Purity	>95 wt %

### Thermophysical properties measurements

**Figure 3:**
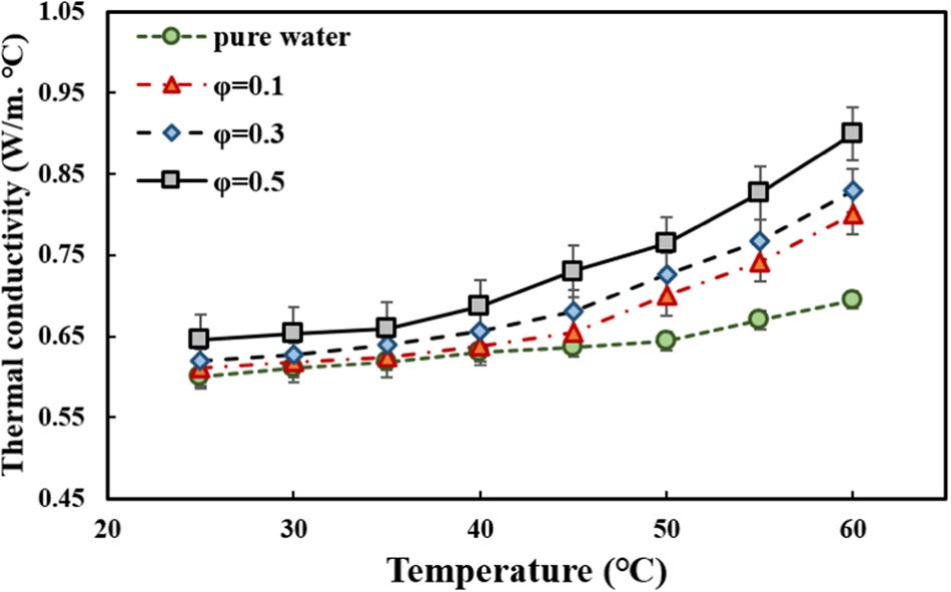
Variations of thermal conductivity of MWCNT-water nanofluid versus temperature and solid concentration, at the optimum ultrasonication time (60 min)^54^.

Ultrasonication time affects the thermophysical properties and HTP of nanofluids^59,60^ and good stability and dispersion of nanoparticles in base fluid results in higher thermal conductivity and lower dynamic viscosity^61,62^. In the authors’ previous work^54^, the thermal conductivity was measured, employing the KD2 Pro thermal analyzer, at different sonication times, temperatures, and solid concentrations. The device was calibrated with pure water, prior to conducting each set of experiments, and the results were compared to the data available in the ASHRAE handbook^63^. The maximum deviation between the experimental data and the ASHRAE handbook was less than ±1.5%. Figure 3 shows the variations of thermal conductivity at the optimum ultrasonication time versus temperature in different solid concentrations. The results showed that the maximum thermal conductivity, which is highly desirable in heat transfer applications, occurred at the ultrasonication time of 60 *min*, solid concentraion of 0.5 *vol*. %, and temperature of 60 °C, was under 30%.

**Figure 4:**
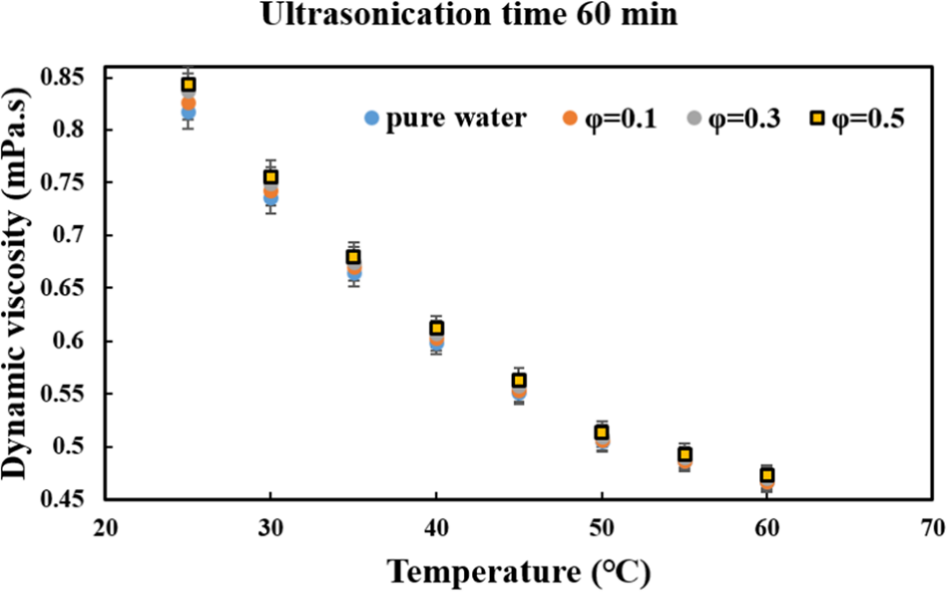
Variations of the dynamic viscosity of MWCNT-water nanofluid versus temperature and solid concentration at the optimum ultrasonication time (60 min).

In this study, the dynamic viscosity of the MWCNT-water nanofluid was measured using a Brookfield cone and plate viscometer (Brookfield laboratory, USA), at different solid concentrations and temperatures. The viscometer was calibrated prior to conducting the measurement, using the glycerol provided by the manufacturer and pure water; the maximum deviation between the measured data and those available in the ASHRAE handbook, was less than ±2%. The viscometer can measure viscosity in the range of 0.0002–15 Pa.s. The accuracy of the viscometer is ±2% and the repeatability of the results is ±5%. The best advantage of using this type of viscometer is the integrated temperature control system, which easily controls the temperature of the samples in the range of 5–75 °C. Moreover, the samples needed for the measurement can be as small as 1 *mL*. It should be noted that the measurements were done at the optimum ultrasonication time of 60 *min*. The variations of dynamic viscosity versus temperature, in different solid concentrations and at the ultrasonication time of 60 *min*, are presented in Fig. 4. The figure shows that the dynamic viscosity decreased as the temperature increased, which is in accordance with the previously published literature^35,36^. The increase in dynamic viscosity by increasing the solid concentrations is witnessed in all the chosen temperatures. However, this increase is not significant. It is interesting to note that the minimum dynamic viscosity, which is highly desirable in fluid dynamics, occurred at a 60 *min* ultrasonication, solid concentration of 0.1 *vol*. %, and temperature of 60 °C, by under 1%, while the maximum increase occurred at the highest solid concentration (0.5 *vol*. %) and the lowest temperature (25 *°C*), by higher than 3%.

## Results and Discussion

In the previous section, the effects of the addition of MWCNT nanoparticles on the thermophysical properties of the base fluid were presented. Adding MWCNT resulted in a negligible increase in the dynamic viscosity and a considerable enhancement in the thermal conductivity of the base fluid. These two parameters affect the HTP and PP; enhancing the thermal conductivity, resulted in enhancing the HTP, and increasing the dynamic viscosity led to an increase in the required PP. In the following sections, the HTP and PP will be evaluated, based on FOMs and theoretical heat transfer equations, in both the fully developed internal laminar and turbulent flow regimes. Moreover, the thermal and fluid dynamic performance of the nanofluid will be evaluated, and the results will be discussed.

### Thermal Effectiveness of MWCNT-water nanofluid

Adding nanoparticles to conventional working fluids results in changing their thermophysical properties. However, the thermophysical properties of a fluid have a direct effect on its HTP. Thus, it is important to have a clear understanding of the HTP of a nanofluid, before conducting experimental studies, to evaluate whether the nanofluid would add an advantage from the heat transfer point of view. To do so, there are FOMs that can evaluate the heat transfer capability of any fluids, based on their thermophysical properties. Asadi^47^ has also proposed a guideline on how to select an effective nanofluid for heat transfer applications, which is based on Prasher *et al*.^48^ and *Mo* number^49^.

#### Fully developed internal laminar flow

**Figure 5:**
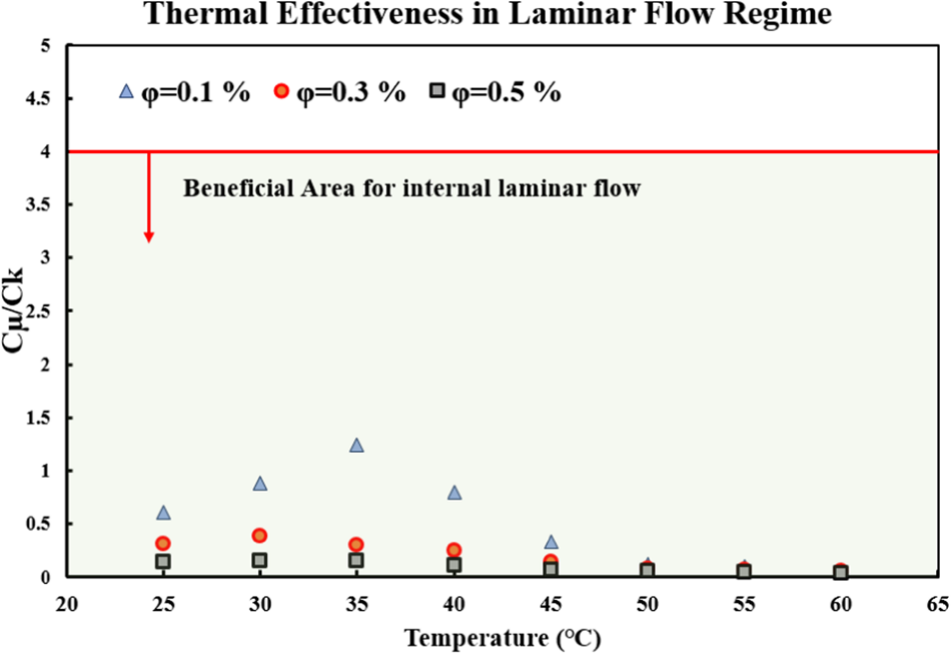
Variations of C_µ_/C_k_ (Prasher *et al*.^48^ FOM) versus temperature in different solid concentrations, in the fully developed internal laminar flow regime.

Prasher *et al*.^48^ proposed a FOM to evaluated the effectiveness of a nanofluid in heat transfer applications, as follows:

1 $$\frac{{C}_{\mu }}{{C}_{\mu }}=\frac{\frac{({\mu }_{nf}-{\mu }_{bf})}{{\mu }_{bf}}}{\frac{({k}_{nf}-{k}_{bf})}{{k}_{bf}}},$$

where *µ* and *k* are the dynamic viscosity and thermal conductivity, respectively. The indices of *nf* and *bf* represent the nanofluid and the base fluid, respectively. It is suggested that the nanofluid may be an effective heat transfer fluid if the ratio of *C*_*µ*_*/C*_*k*_ is less than four. This means that the increase in dynamic viscosity should be less than four times that of the thermal conductivty of the nanofluid else, the nanofluid may not be a good heat transfer fluid. Figure 5 shows the variations of *C*_*µ*_*/C*_*k*_ versus temperature, in different solid concentrations. As can be seen, *C*_*µ*_*/C*_*k*_ is much less than 4 for the various temperatures and solid concentrations. Thus, it can be concluded that the studied nanofluid would be a good heat transfer fluid in the range of the studied temperatures and solid concentrations, in a fully developed internal laminar flow regime.

Another FOM to evaluate the HTP of a fluid is the *Mo* number^49^, which can be used in both the fully developed internal laminar and turbulent flow regimes:

2 $$Mo={\rho }^{a}{k}^{b}{c}_{p}^{c}{\mu }^{-d},$$

where *ρ* represents density, *k* is thermal conductivity, *c*_*p*_ is specific heat capacity, and *µ* represents the dynamic viscosity. The values of exponents *a, b, c*, and *d* vary, depending on the heat transfer mode of interest^64^. In the internal laminar flow regime, the Nusselt (Nu) number is a constant value and thermal conductivity is the only effective parameter on the CHTC^47^. Thus, the relative thermal conductivity (*k*_*nf*_*/k*_*bf*_) is equal to the ratio of *Mo* numbers, as follows:

3 $$\frac{M{o}_{nf}}{M{o}_{bf}}=\frac{{k}_{nf}}{{k}_{bf}}.$$

**Figure 6:**
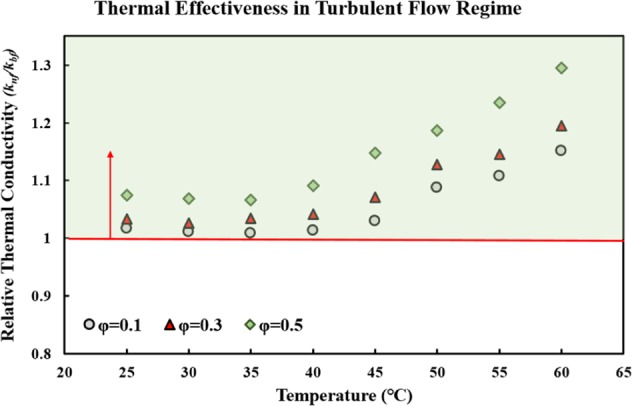
Variations of the relative thermal conductivity of MWCNT-water nanofluid versus temperature for different solid concentrations.

If the ratio of *Mo*_*nf*_/*Mo*_*bf*_ (*k*_*nf*_/*k*_*bf*_ = relative thermal conductivity) is higher than 1, the nanofluid is a good heat transfer fluid for the internal laminar flow regime. Figure 6 displays the variations of the relative thermal conductivity of MWCNT-water nanofluid versus temperature in different solid concentrations. As seen in the figure, the value of the relative thermal conductivity was higher than 1 for all the temperatures and solid concentrations. Moreover, an increase in the temperature and solid concentration led to an increase in the value of the relative thermal conductivity. Thus, it would be concluded that using the studied nanofluid, instead of the base fluid, provides certain heat transfer advantages in the fully developed internal laminar fluid regime.

#### Fully developed internal turbulent flow

As stated in the previous section, the *Mo* number^49^ is a good FOM to evaluate the HTP of a fluid, in both the laminar and turbulent flow regimes. In the turbulent flow regime, the *Mo* number^49^ is expressed as follows:

4 $$Mo={\rho }^{0.8}{k}^{0.67}{c}_{p}^{0.33}{\mu }^{-0.47}.$$

The ratio of *Mo*_*nf*_/*Mo*_*bf*_ should be higher than 1 in this case as well, to indicate that the fluid is a good heat transfer fluid; the higher the ratio, the better the performance of the nanofluid. It is important to note that the density and specific heat of the nanofluid was calculated, based on the correlation presented by Pak and Cho^65^:

5 $${\rho }_{nf}=(1-\varphi ){\rho }_{bf}+\varphi {\rho }_{Particle}$$

6 $${c}_{p,nf}=(1-\varphi ){c}_{p,bf}+\varphi {c}_{p,Particle}.$$

**Figure 7:**
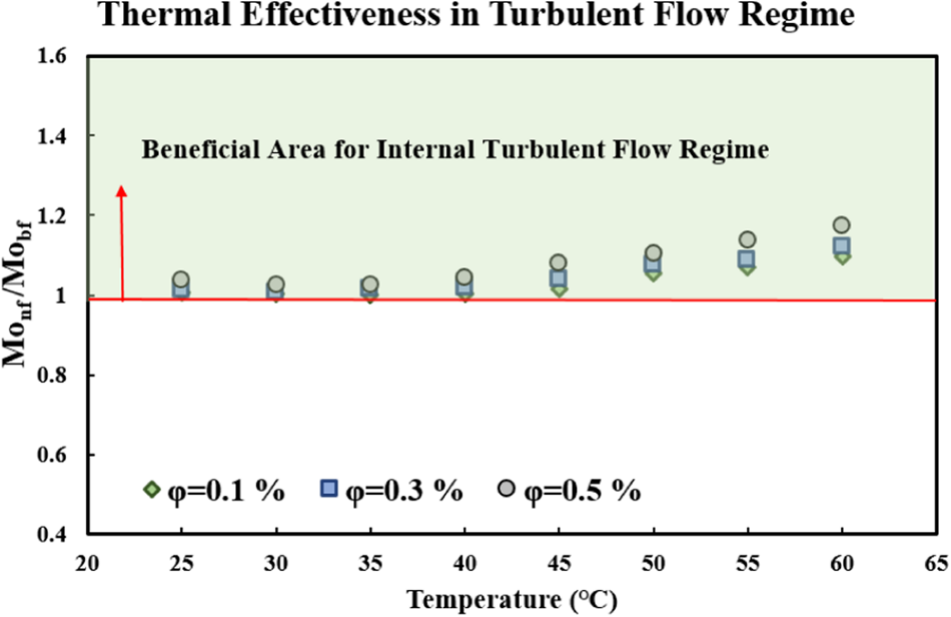
Variations of Mo_nf_/Mo_bf_ of the studied nanofluid versus temperature for different solid concentrations, in the internal turbulent flow regime.

Figure 7 shows the variations of *Mo*_*nf*_*/Mo*_*bf*_ versus temperature, for different solid concentrations. As seen in the figure, the ratio of *Mo*_*nf*_*/Mo*_*bf*_ was higher than 1 for all the studied temperatures and solid concentrations, and an increase in the temperature and solid concentrations led to an increase in the ratio of *Mo*_*nf*_*/Mo*_*bf*_. It can thus be concluded that the studied nanofluid is a potential heat transfer fluid, in the fully developed internal turbulent flow regime.

### Pumping power

It was previously mentioned that adding MWCNT particles to water leads to an increase in the dynamic viscosity of the resultant fluid. Increasing the dynamic viscosity also leads to an increase in the PP required to supply the same fluid velocity. Thus, it is important to assess the increase in energy consumption, by increasing the PP in a system. With regard to this, Mansour *et al*.^66^ proposed a FOM to evaluate the PP for the internal laminar and turbulent flow regimes, which will be presented and discussed in the following sections.

#### Internal laminar flow

For the internal laminar flow regime, the increase in PP can be evaluated as Mansour *et al*.^66^ suggested:

7 $$\frac{{W}_{nf}}{{W}_{bf}}=\left(\frac{{\mu }_{nf}}{{\mu }_{bf}}\right).{\left(\frac{{\rho }_{bf}}{{\rho }_{nf}}\right)}^{2}.$$

**Figure 8:**
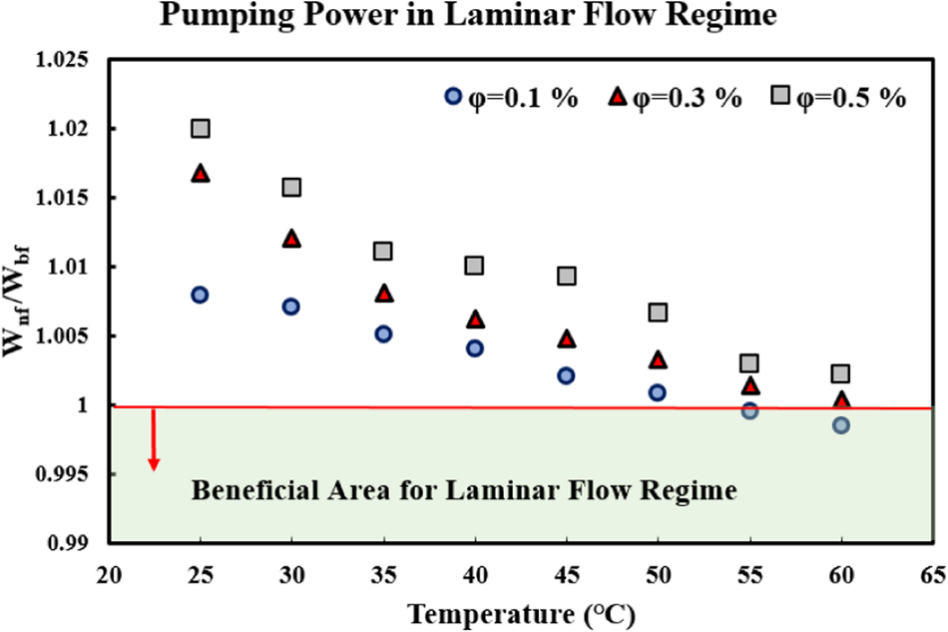
The effects of using MWCNT-water nanofluid on the increase of pumping power, in the internal laminar flow regime.

From the energy management point of view, the ratio of *W*_*nf*_ */W*_*bf*_ < 1 is advantageous, that is, using the nanofluid instead of the base fluid would not incur a large penalty in PP and energy consumption. However, the heat transfer enhancement should also be evaluated, and then a better assessment can be made. Figure 8 displays the variations of *W*_*nf*_ */W*_*bf*_ versus temperature, for different solid concentrations, for the internal laminar flow regime. As seen in the figure, the ratios are higher than 1 for all the temperatures and solid concentrations, except for the solid concentration of 1 *vol*. % and temperatures of 55 and 60 *°C*. Thus, using the studied nanofluid is not recommended in the internal lamina flow regime, from an energy management point of view.

#### Internal turbulent flow

The suggested FOM by Mansour *et al*.^66^ for evaluating the PP in internal turbulent flow regime is as follows:

8 $$\frac{{W}_{nf}}{{W}_{bf}}={\left(\frac{{\mu }_{nf}}{{\mu }_{bf}}\right)}^{0.25}.{\left(\frac{{\rho }_{bf}}{{\rho }_{nf}}\right)}^{2}.$$

**Figure 9:**
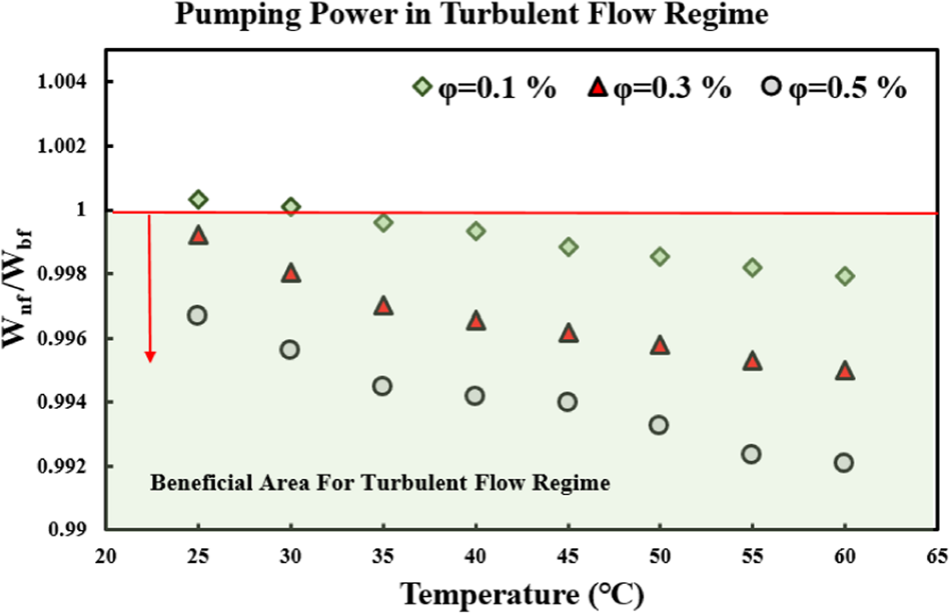
The effects of using MWCNT-water nanofluid on the increase of pumping power, in the internal turbulent flow regime.

A ratio less than 1 is advantageous, from the energy management point of view, for this case as well. Figure 9 presents the possible effects of using MWCNT-water nanofluid, instead of the pure water, on the PP. As seen in the figure, all the ratios are less than 1, similar to the internal laminar flow regime, in all the studied temperatures and solid concentrations, except the solid concentration of 1 *vol*. % and temperatures of 25 and 30 °C. Thus, it can be concluded that employing the studied nanofluid would not require extra PP from the system.

### Thermal and fluid dynamic performance of MWCNT-water Nanofluid

**Figure 10:**
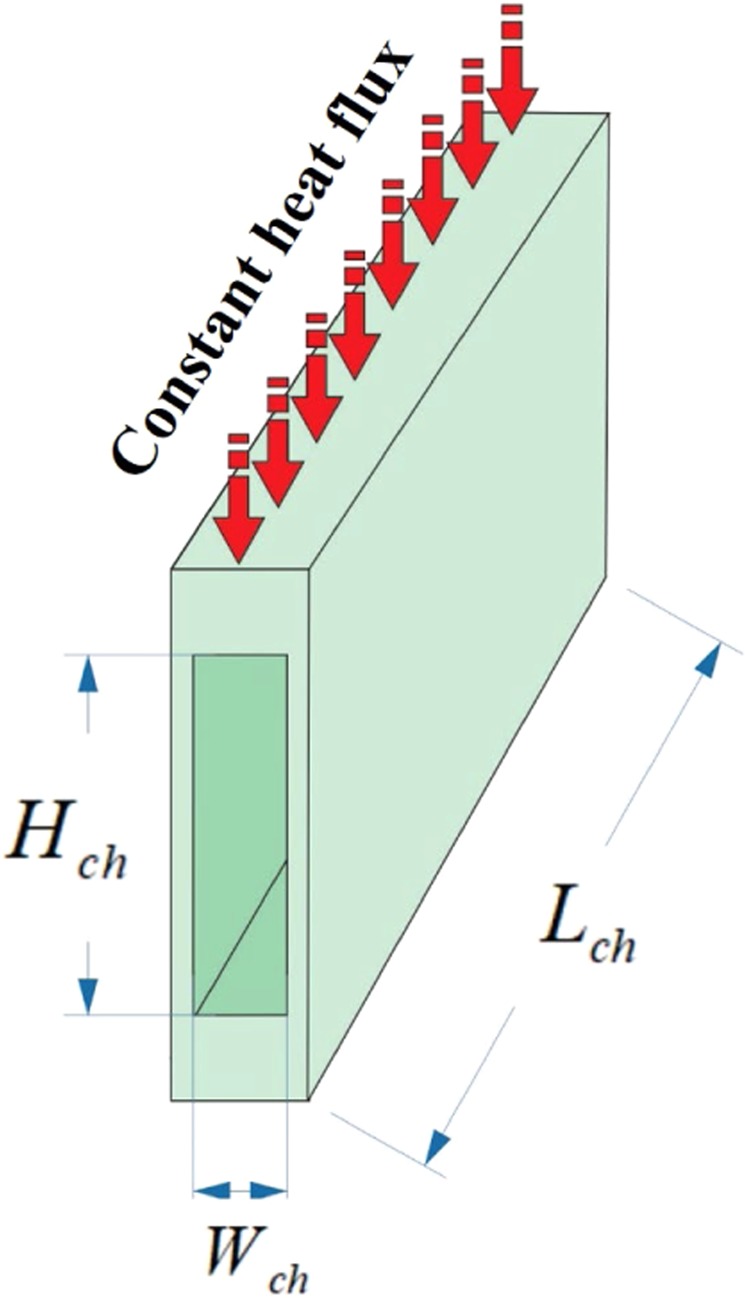
A schematic view of the studied MCHS.

It was shown in the previous sections that adding MWCNT particles to water affects its thermophysical properties, HTP, and PP, in both the internal laminar and turbulent flow regimes, in different temperatures and solid concentrations. It was proven that using the MWCNT-water nanofluid would be beneficial for both the flow regimes. In the following section, the thermal and fluid dynamic performance of the nanofluid was studied, to clearly present the benefits of using MWCNT-water, compared to pure water. To do this, the relative CHTC of the nanofluid in a MCHS, with the dimensions of *W*_*ch*_ = 500 *µm*, *H*_*ch*_ = 1000 *µm*, and *L*_*ch*_ = 20 *mm*, under the laminar flow regime, was investigated. It was assumed that the flow was fully developed, and there was a constant heat flux on the top wall of the MCHS. Figure 10 displays a schematic view of the geometry of the assumed MCHS.

Various correlations have been proposed thus far to calculate the Nu number in microchannels using nanofluids^67–70^. However, none of the models can be used to estimate the Nu number in different microchannels, subjected to different working fluids. However, the main aim of this study is to present a clear overview of using MWCNT-water nanoflui as a potential heat transfer fluid in heat transfer applications. Thus, to calculate the CHTC for the assumed microchannel and boundary conditions, a conventional heat transfer equation has been employed. In the case of a fully developed internal laminar flow, under a constant heat flux, the Nu number can be assumed to be constant. Moreover, the Nu number is dependent only on the microchannel aspect ratio^71–73^, which is equal to 2 (*α*_*ch*_ = *H*_*ch*_*/W*_*ch*_ = 2). Thus the Nu number can be calculated as follows^73^:

9 $$Nu=8.235(1-10.6044{\alpha }_{ch}+61.1755{\alpha }_{ch}^{2}-155.1803{\alpha }_{ch}^{3}+176.9203{\alpha }_{ch}^{4}-72.923{\alpha }_{ch}^{5})$$

The CHTC can then be calculated as follows:

10 $$\begin{array}{c}h=\frac{k}{{D}_{h}}Nu\\ {D}_{h}=\frac{2{W}_{ch}{H}_{ch}}{({W}_{ch}+{H}_{ch})}\end{array}$$

**Figure 11:**
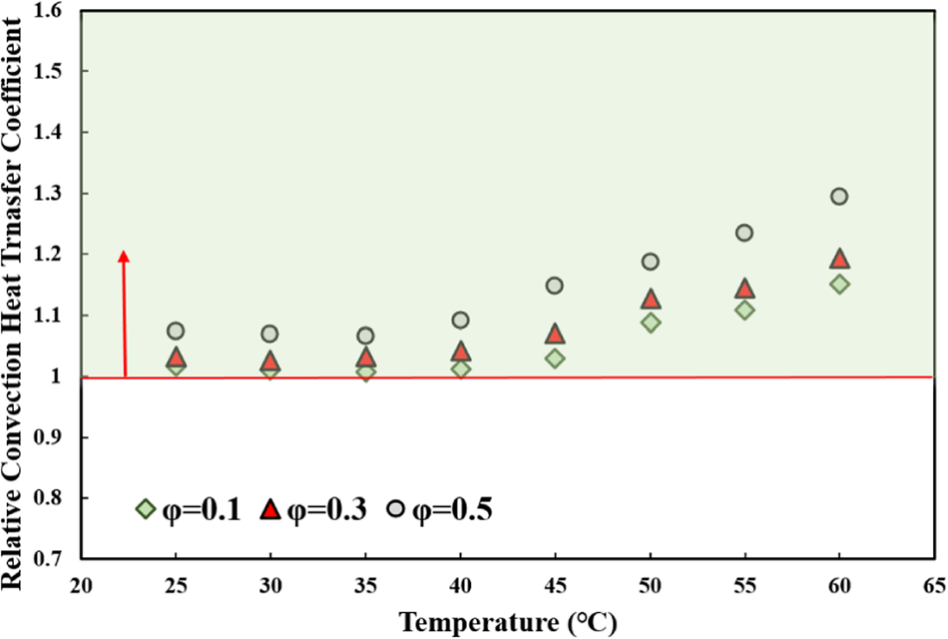
The variations of CHTC versus temperature for different solid concentrations, for the case of fully developed internal laminar flow regime.

Figure 11 shows the effects that adding MWCNT to water had on the CHTC versus temperature relationship, for different solid concentrations. As seen in the figure, the relative CHTC increased as the temperature and solid concentration increased. The increase was more noticeable at temperatures higher than 45 *°C* and solid concentration of 0.5 *vol*. %. It was noted that the highest increase occurred at the solid concentration of 0.5 *vol*. % and temperature of 60 *°C* by under 30%; the lowest increase occurred at the solid concentration of 0.1 *vol*. % and temperature of 35 *°C* by less than 1%. It can be concluded that the studied nanofluid would be a good replacement for the base fluid, especially at high temperatures and solid concentrations. In the following section, the heat transfer increase will be compared to the increase in PP, to reach a firm conclusion of the advantages of using the studied nanofluid in heat transfer applications.

**Figure 12:**
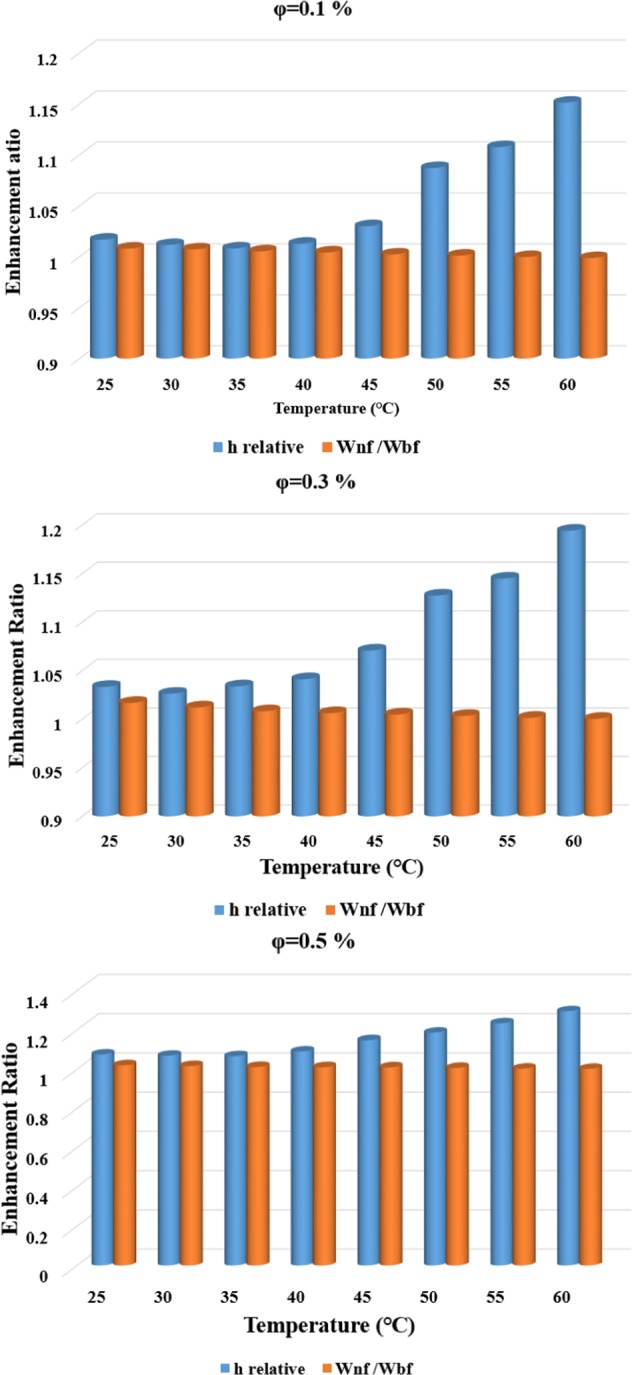
The thermal and fluid dynamic performance of MWCNT-water nanofluid in the studied range of temperatures and solid concentrations.

It is important to evaluate the possible benefits of using nanofluids because they are more expensive than conventional working fluids, and thermal and fluid dynamic performance is one of the reliable ways to evaluate the new fluid. In this study, the thermal and fluid dynamic performance of MWCNT-water nanofluid was evaluated over the studied range of solid concentrations and temperatures. This was done to assess the effectiveness of the nanofluid, by comparing the enhancement ratio of the CHTC and PP. Figure 12 presents the thermal and fluid dynamic performance of the studied nanofluid, for different temperatures and solid concentrations. The figure shows that at the solid concentration of 0.1 *vol*. % and temperatures less than 45 *°C*, there was a negligible difference in the enhancement ratio of the CHTC and the increase in PP. However, increasing the temperature to higher than 45 °C led to a significant difference between the enhancement ratio of the CHTC and the increase in PP. This trend was similar in solid concentrations of 0.3 and 0.5 *vol*. %, with some difference in the solid concentration of 0.5 *vol*. %. The difference between the enhancement ratio of the CHTS and the increase in the PP was negligible, compared to the other two solid concentrations. It is important to note that the best performance was observed at the solid concentration of 0.3 *vol*. %. It can thus be concluded that using MWCNT-water nanofluid, instead of pure water, is advantageous in heat transfer applications while imposing a negligible penalty in PP.

## Conclusion

In this study, the thermal and fluid dynamic performance of MWCNT-water nanofluid was investigated, based on the thermophysical properties of the nanofluid. The study was done over a range of different temperatures and solid concentrations. Moreover, the effects that applying different ultrasonication times had on the stability and thermophysical properties of the nanofluid were investigated. The results of the zeta potential and visual observation, over 30 days after sample preparations, revealed that 60 *min* ultrasonication was the optimum time for which the samples possessed the most stability. It was also revealed that the dynamic viscosity reached its minimum increase by applying a 60 *min* ultrasonication. The HTP of the nanofluid was investigated, using the experimentally obtained thermophysical properties data, and employing different FOMs. The nanofluid was a good heat transfer fluid, in both the fully developed internal laminar and turbulent flow regimes, over the studied range of temperatures and solid concentrations. Moreover, the HTP of the nanofluid in a MCHS, under the constant heat flux boundary condition, was studied; the study showed that the nanofluid would increase the heat transfer by a maximum of 30%. The effects of the addition of MWCNT on PP was also studied, and it was found that it had a negligible effect. Finally, the thermal and fluid dynamic performance of the nanofluid were investigated, by comparing the enhancement ratio of PP versus heat transfer, in the studied range of temperatures and solid concentrations. The ratio of the heat transfer enhancement was higher than the increase in the PP. Thus, the studied nanofluid would be a good heat transfer fluid in heat transfer applications, with a minimum penalty in energy consumption.
